# Analysis of **LMNB*1* Duplications in Autosomal Dominant Leukodystrophy Provides Insights into Duplication Mechanisms and Allele-Specific Expression

**DOI:** 10.1002/humu.22348

**Published:** 2013-05-28

**Authors:** Elisa Giorgio, Harshvardhan Rolyan, Laura Kropp, Anish Baswanth Chakka, Svetlana Yatsenko, Eleonora Di Gregorio, Daniela Lacerenza, Giovanna Vaula, Flavia Talarico, Paola Mandich, Camilo Toro, Eleonore Eymard Pierre, Pierre Labauge, Sabina Capellari, Pietro Cortelli, Filippo Pinto Vairo, Diego Miguel, Danielle Stubbolo, Lourenco Charles Marques, William Gahl, Odile Boespflug-Tanguy, Atle Melberg, Sharon Hassin-Baer, Oren S Cohen, Rastislav Pjontek, Armin Grau, Thomas Klopstock, Brent Fogel, Inge Meijer, Guy Rouleau, Jean-Pierre L Bouchard, Madhavi Ganapathiraju, Adeline Vanderver, Niklas Dahl, Grace Hobson, Alfredo Brusco, Alessandro Brussino, Quasar Saleem Padiath

**Affiliations:** 1 University of Torino, Department of Medical SciencesTorino, Italy; 2 Department of Human Genetics Graduate School of Public Health, University of PittsburghPittsburgh, Pennsylvania; 3 Department of Biomedical Informatics School of Medicine, University of PittsburghPittsburgh, Pennsylvania; 4 Department of Obstetrics Gynecology and Reproductive Sciences, University of PittsburghPittsburgh, Pennsylvania; 5 Department of Pathology University of Pittsburgh, School of MedicinePittsburgh, Pennsylvania; 6 S.C.D.U. Medical Genetics, Az. Osp. Città della Salute e della ScienzaTorino, Italy; 7 Department of Neuroscience, Az. Osp. Città della Salute e della ScienzaTorino, Italy; 8 Department of Neurology, Ophthalmology and Genetics, di Bologna, Department of Biomedical and NeuroMotor Sciences (DIBINEM) Alma Mater StudiorumBologna, Italy; 9 NIH Undiagnosed Diseases Program NIH Office of Rare Disease, Research and NHGRIBethesda, Maryland; 10 CHU de Clermont-Ferrand, Department of Genetics and CytogeneticsFrance; 11 Neurologie Hopital Caremeau, Centre Hospitalo-Universitaire de NimesNimes, France; 12 University of Bologna IRCCS Istituto delle Scienze Neurologiche di Bologna Department of Biomedical and NeuroMotor Sciences (DIBINEM), Alma Mater StudiorumItaly; 13 Hospital de Clínicas de Porto Alegre … Universidade Federal do Rio Grande do SulPorto Alegre, Brazil; 14 Nemours Biomedical Research, Alfred I. duPont Hospital for ChildrenWilmington, Delaware; 15 Department of Medical Genetics Clinics Hospital of Ribeirao Preto, University of Sao PauloSao Paulo, Brazil; 16 Institut National de la Santé et de la Recherche Médicale (INSERM) – Paris Diderot Sorbonne Paris Cité University, Robert Debré HospitalParis, France; 17 Assistance Publique des Hopitaux de Paris Reference Center for Rare Diseases “Leukodystrophies”, Child Neurology and Metabolic Disorders DepartmentParis, France; 18 Department of Neuroscience Neurology, Uppsala UniversityUppsala, Sweden; 19 Parkinson’s disease and Movement Disorders Clinic Department of Neurology, Chaim Sheba Medical CenterTel Aviv, Israel; 20 Sackler Faculty of Medicine, Tel Aviv UniversityTel Aviv, Israel; 21 Department of Neurology, University of HeidelbergHeidelberg, Germany; 22 Dept. of Neurology, Klinikum LudwigshafenLudwigshafen, Germany; 23 Dept. of Neurology Friedrich-Baur-Institute, Ludwig-Maximilians-UniversityMunich, Germany; 24 German Center for Vertigo and Balance DisordersMunich, Germany; 25 DZNE – German Center for Neurodegenerative DiseasesMunich, Germany; 26 German Network for Mitochondrial Disorders(mitoNET), Germany; 27 Department of Neurology David Geffen School of Medicine, University of CaliforniaLos Angeles, California; 28 Montreal Neurological Institute, McGill UniversityMontreal, Canada; 29 Department of Neurological Sciences, CHA – Hôpital de l’Enfant-JésusQuebec City, Canada; 30 Department of Neurology, Childrens National Medical CenterWashington, District of Columbia; 31 Dept. of Immunology Genetics and Pathology Section of Clinical Genetics The Rudbeck laboratory, Uppsala University Children’s HospitalUppsala, Sweden; 32 University of Delaware, Department of BiologyNewark, Delaware; 33 Thomas Jefferson University, Jefferson Medical CollegePhiladelphia, Pennsylvania

**Keywords:** Lamin B1, leukodystrophy, ADLD, duplication Alu, NHEJ, FoSTeS, MMBIR

## Abstract

Autosomal dominant leukodystrophy (ADLD) is an adult onset demyelinating disorder that is caused by duplications of the lamin B1 (**LMNB*1*) gene. However, as only a few cases have been analyzed in detail, the mechanisms underlying **LMNB*1* duplications are unclear. We report the detailed molecular analysis of the largest collection of ADLD families studied, to date. We have identified the minimal duplicated region necessary for the disease, defined all the duplication junctions at the nucleotide level and identified the first inverted **LMNB*1* duplication. We have demonstrated that the duplications are not recurrent; patients with identical duplications share the same haplotype, likely inherited from a common founder and that the duplications originated from intrachromosomal events. The duplication junction sequences indicated that nonhomologous end joining or replication-based mechanisms such fork stalling and template switching or microhomology-mediated break induced repair are likely to be involved. **LMNB*1* expression was increased in patients’ fibroblasts both at mRNA and protein levels and the three **LMNB*1* alleles in ADLD patients show equal expression, suggesting that regulatory regions are maintained within the rearranged segment. These results have allowed us to elucidate duplication mechanisms and provide insights into allele-specific **LMNB*1* expression levels.

## Introduction

Adult-onset autosomal dominant leukodystrophy (ADLD) is a rare demyelinating disease with an onset in the fourth or fifth decade of life. The clinical presentation usually consists of initial autonomic symptoms followed by pyramidal signs and ataxia [Lin et al., 2011; Padiath and Fu, 2010]. Cardiovascular and skin noradrenergic failure was recently found in one ADLD family, and might be another hallmark of the disease [Guaraldi et al., 2011]. Magnetic resonance imaging showed diffuse and symmetrical supra- and infratentorial white matter changes, particularly of cerebellum, corticospinal tracts, and corpus callosum. Some patients showed brain and spinal cord atrophy [Sundblom et al., 2009]. Histological evaluation of brain lesions displays astrogliosis and oligodendrocyte preservation [Coffeen et al., 2000; Melberg et al., 2006].

ADLD was shown to be caused by a duplication involving the lamin B1 gene (*LMNB1*; MIM #150340), on chr. 5q32. The duplication resulted in an increased expression of lamin B1 mRNA and protein in patient brain tissue [Padiath et al., 2006]. ADLD thus joins a growing list of neurological diseases caused by changes in gene copy number. These include Pelizaeus–Merzbacher Disease (PMD), caused by duplications of the proteolipid 1 protein (*PLP1*; MIM #300401) and developmental delay with intellectual disability caused by duplications of the methyl-CpG-binding protein 2 gene (*MECP2*; MIM #300005) [Lee and Lupski, 2006; Stankiewicz and Lupski, 2010].

After the initial identification of the *LMNB1* duplication in three independent ADLD families, sporadic reports of single ADLD families from different parts of the world were published [Brussino et al., 2009a; 2009b; Dos Santos et al., 2012; Fogel et al., 2012; Meijer et al., 2008; Padiath and Fu, 2010; Schuster et al., 2011]. However, in all these reports, only approximate duplication boundaries were determined.

Apart from the characterization of the duplication junction sequences in two patients in the initial report that identified *LMNB1* duplications, no further *LMNB1* duplication junctions have been resolved at the base pair level [Padiath et al., 2006; Padiath and Fu, 2010]. The analysis of duplication junction sequences is essential for understanding the molecular mechanisms that give rise to such events. Nonallelic homologous recombination (NAHR) and non homologous end joining (NHEJ) have been proposed for the generation of both normal and pathogenic copy number variations [Hastings and Rosenberg, 2011; Stankiewicz and Lupski, 2010; van Binsbergen, 2011; Woodward et al., 2005]. Replication-based mechanisms such Fork stalling and template switching (FoSTeS) and microhomology-mediated break induced repair (MMBIR) have also been implicated [Hastings et al., 2009; Stankiewicz and Lupski, 2010].

In this report, we describe, to the best of our knowledge, the analysis and systematic molecular characterization of the largest collection of ADLD patients with *LMNB1* duplications presently available.

## Methods

### Patients

DNA samples were obtained from 31 ADLD patients from 20 independent families from different laboratories worldwide (USA *n* = 8, Italy *n* = 5, Sweden *n* = 4, Germany *n* = 4, France *n* = 3, India *n* = 3, Canada *n* = 2, Israel *n* = 1, Brazil *n* = 1). Nine of the 20 families have been described previously (Supp. Table S1). The remaining families were screened at the A.I. duPont Hospital for Children, University of Torino, UCLA, Children’s National Medical Center and the University of Pittsburgh. All studies were carried out after obtaining ethical approval from the institutional review boards of the respective institutions. DNA was extracted from blood or cell lines using the Puregene DNA isolation kit or the Qiamp blood kit (Qiagen, Mannheim, Germany). All families were screened based on clinical symptoms consistent with the ADLD phenotype. Fibroblast cell lines were available for six patients (IT1, IT2, IT3, A2, A3, BR1). PAXgene-stabilized blood sample for RNA isolation (Qiagen) was obtained from one patient (IT3–1).

### Custom Array Comparative Genomic Hybridization and Breakpoint Identification

To define the boundaries of the duplications, two custom 8 × 15K array Comparative Genomic Hybridization (aCGH) assays were designed using the eArray tool (https://earray.chem.agilent.com/earray/, Agilent Technologies Inc., Palo Alto, CA). Array CGH assays carried out at the University of Torino had an average probe spacing of ∼800 bp between 125,010,000 and 127,269,000 Mb on chromosome 5. Arrays at the University of Pittsburgh had an average probe spacing of ∼200 bp between positions chr5:125,112,315 and chr5:127,172,712. Experiments were performed following manufacturer’s instructions and the slides scanned on either a G2565BA or G2565CA scanner and analyzed using Agilent CGH Analytics software ver.5.0.14 or the Agilent Cytogenomics software 2.0.6.0 (Agilent Technologies Inc.). Duplication breakpoints were identified by PCR amplification with different combination of primers in each patient (Supp. Table S2) using the KAPA2G Fast PCR kit (Kapa Biosystems, Inc., Woburn, MA), or the New England Biolabs Long PCR kit (NEB, Ipswich, MA) following manufacturer’s instructions. Control samples were also used in the long PCR reactions to confirm that amplification occurred only in patient samples.

Inverse PCR was performed on the genomic DNA derived from the patient in BR1 family to identify duplication breakpoints using a protocol described previously using either *Rsa*I or *Bgl*II restriction endonucleases (primers used are listed in Supp. Table S2).

### Bioinformatics Analyses

All sequences and sequence coordinates were obtained from the UCSC genome browser (assembly GRCh37/hg19) and are from chromosome 5. For the majority of the analyses, we selected a 200 bp region surrounding the centromeric and telomeric duplication breakpoint sequences (referred to as patient breakpoint sequences) and compared them with 500 sequences of 200 bp (referred to as control sequences) randomly selected from chromosome 5 using an approach similar to that described previously [Carvalho et al., 2009; Vissers et al., 2009]. In the control sequences, the breakpoint was arbitrarily defined as sequence between base 100 and 101 (the middle of the sequence). We calculated the percentage of simulated breakpoints that fell within repetitive elements. For GC% analysis, we used a 4 kb region surrounding the patient duplication breakpoints and 500 4-kb random sequences from chromosome 5.

The following online tools were used to compare the patient breakpoints and control sequences: RepeatMasker (http://www.repeatmasker.org/), GEECEE (http://150.185.138.86/cgi-bin/emboss/geecee), Fuzznuc (http://150.185.138.86/cgi-bin/emboss/fuzznuc) [Vissers et al., 2009], MEME (http://meme.sdsc.edu/meme/intro.html), and the database Non-B DB (http://nonb.abcc.ncifcrf.gov/apps/site/default). Low copy repeats (LCRs) were identified using the segmental duplication track on the UCSC genome browser (http://www.genome.ucsc.edu).

### Primer Extension Assay

To verify if one of the two *LMNB1* alleles was preferentially expressed in duplication carriers, we evaluated the relative amount of the two alleles of a heterozygous SNP (rs#1051644, c.*239C>T) in the 3′UTR of *LMNB1* using a primer extension assay with the SNaPshot System (Applied Biosystems, Foster City, CA).

Two reference plasmid clones, one for each rs#1051644 allele, were prepared to build a reference curve. We amplified a 497 bp fragment centered on the SNP with 500 nM primers 5′-aaagggtccatttgaggttagg and 5′-tggtttatttaccctcccctcct from a heterozygous control.

The PCR product was gel purified with the HiYeld™ Gel/PCR Fragments Extraction Kit (RBC Bioscience, Taipei, Taiwan), inserted into a pTZ57R/T plasmid using the TA Cloning Kit (Invitrogen/Life technologies, Grand Island, NY). They were sequence verified to isolate one clone for each allele (pTZ57R/T_*LMNB1*_c.*239C and pTZ57R/T_*LMNB1*_c.*239T) and quantified on a Qubit instrument (Invitrogen/Life Technologies). Mixes were prepared with proportions of 35%, 50%, and 65% C/T alleles and used to obtain a standard curve.

Primer extension was performed amplifying from genomic DNA with the primers and conditions reported above. On cDNA, we amplified a 394 bp fragment using primers 5′-gaagaacttttccaccagcag and 5′-tggtttatttaccctccctcct. PCR products were purified using Exonuclease I and Shrimp Alkaline Phosphatase (SAP, MBI-Fermentas, Vilnius, Lithuania) and the primer extension reaction performed with primers annealing immediately before and after the SNP base (5′-gactgactctgaacttaataactgtgtactgtt, 5′-ctgactgactgacttgaggaaccccttcc). SNaPshot reactions were purified using Shrimp Alkaline Phosphatase (SAP), loaded on an ABI-Prism 3100 Avant capillary electrophoresis instrument with a GS120-Liz marker and analyzed using the GeneScan ver 3.7 software (Applied Biosystems).

Additional methods are available in the Supporting Information.

*LMNB1* gene variants have been submitted to the Leiden Open Variation Database (www.lovd.nl/LMNB1)

## Results

### Characterization of ADLD Duplications

**Figure 1:**
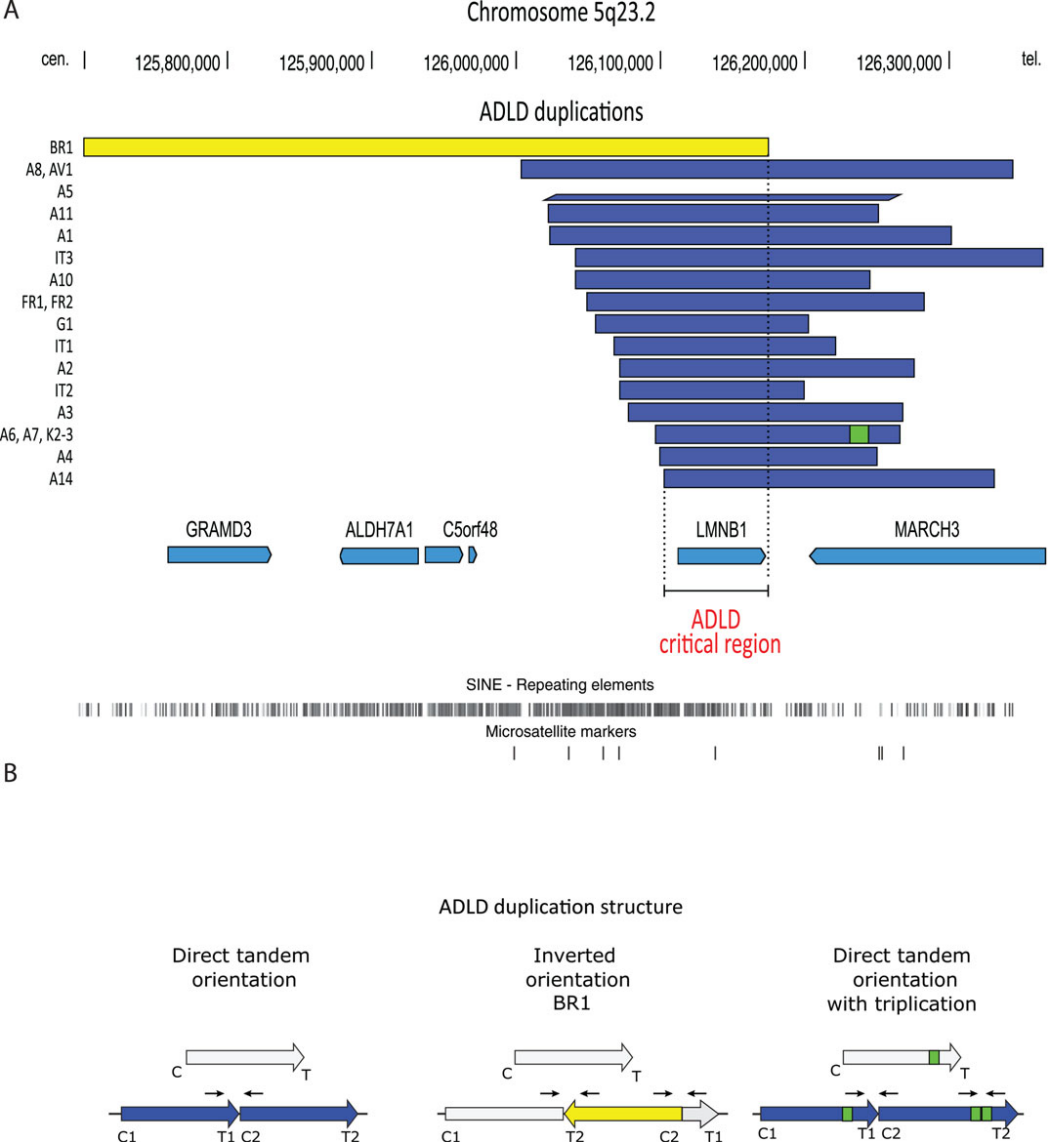
Overview of the genomic rearrangements in the ADLD families. A: Modified output from the UCSC genome browser showing the *LMNB 1* gene duplications in 20 ADLD families (16 unique duplications) and their surrounding genomic region. The duplications are marked in blue, with the exceptions of the BR1 duplication/inversion, which is in yellow and the triplicated segment, which is in green. Duplications marked with asterisks (*) have sequence insertions at their duplication junctions and show a clustering of their centromeric breakpoints within a 25 kb segment. The minimal critical region duplicated in ADLD of ∼75 kb is also shown. The location of SINE repetitive elements and microsatellite markers used in genotyping (modified UCSC genome browser tracks) are shown below. Note the enrichment of SINE elements (the majority of which are *Alu* repetitive elements) centromeric to the *LMNB 1* gene. B: Schematic representation of the three *LMNB1* duplication configurations identified. C1–T1 and C2–T2 represent the duplicated segments that are derived from the parental genomic region, C–T. Black arrows represent orientation of primers used to for PCR and sequencing across duplication and triplication junctions.

We collected 20 independent ADLD families, in which genomic *LMNB1* duplication was initially identified by aCGH, QT-PCR or Multiplex Ligation-dependent Probe Amplification (Table 1). Approximate duplication boundaries have previously been described in seven cases [Brussino et al., 2009a; Dos Santos et al., 2012; Meijer et al., 2008; Schuster et al., 2011]. Using a custom array encompassing 2 Mb around the *LMNB1* gene, we were able to accurately determine all duplication sizes, finding a total of 16 unique rearrangements (Fig. 1A and B, Table 1). Three of the duplications were shared by more than one family (Table 1): one was found in three families (A6, A7, and K2–3) and the other two in two families each (A8 and AV1, FR1 and FR2).

**Table 1:** Details of the 16 Unique **LMNB*1* Duplications

	Family #	Size	Centromeric breakpoint	Telomeric breakpoint	Nature of junction	Repetitive element at duplication breakpoints		Possible mechanism
						Centromeric	Telomeric	
1	Al	277,929	126,023,423	126,301,352	Insertion of “GCAC”	–	–	NHEJ
2	A2	203,432	126,072,067	126,275,499	Microhomology of “T”	–	MER82	NHEJ/FoSTeS-MMBIR
3	A3	189,731	126,078,035	126,267,766	Microhomology of “AC”	–	–	NHEJ/FoSTeS-MMBIR
4	A4	150,283	126,099,593	126,249,876	19 bp Homology of AluY element	AluY	AluY	NAHR/FoSTeS-MMBIR
5	A5	238,946	126,018,887	126,257,833	Microhomology of “AAGGGA”	–	–	NHEJ/FoSTeS-MMBIR
6	A6, A7, K2-3	169,456	126,096,876	126,266,332	Microhomology of “CT”	–	–	NHEJ/FoSTeS-MMBIR
6*	A6, A7, K2-3	13,656	126,230,827	126,244,483	146 bp Homology of LINE element	LIP A3	LIP A3	NAHR
7	A8, AV1	340,785	126,003,283	126,344,068	Microhomology of “AC”	LTR7B	–	NHEJ/FoSTeS-MMBIR
8	A10	203,842	126,041,308	126,245,150	Insertion of CTAGTG	LTR78B	LIP A3	NHEJ
9	All	228,672	126,022,573	126,251,245	Microhomology of “GG”	–	–	NHEJ/FoSTeS-MMBIR
10	A14	229,243	126,102,443	126,331,686	Microhomology of GA	AluSg	L2c	NHEJ/FoSTeS-MMBIR
11	Gl	148,085	126,054,572	126,202,657	Microhomology of “CAG”	–	–	NHEJ/FoSTeS-MMBIR
12	FR1,FR2	234,020	126,049,232	126,283,252	Insertion of TAGCTAAGTTA	L1MB7	L1MC1	NHEJ
13	IT1	153,769	126,068,010	126,221,779	Microhomology of “AA”	AluSx		NHEJ/FoSTeS-MMBIR
14	IT2	127,608	126,072,145	126,199,753	Microhomology of “GCTG”	–		NHEJ/FoSTeS-MMBIR
15	IT3	324,675	126,040,794	126,365,469	Insertion of “ATGTTTGTATTT”	AluSx	–	NHEJ
16	BR1	474,998	125,699,519	126,174,517	Complex	LIMB 7	–	FoSTeS-MMBIR

*Note:* Coordinates refer to chromosome 5, February 2009 assembly of the reference genome (GRCh37/hg19), Asterisk

(^*^) indicates triplication.

Duplication sizes ranged from ∼128 to ∼475 kb, which represent the smallest and largest ADLD duplications so far identified. The largest duplication, found in the patient from the BR1 family, also included the *PHAX*, *ALDH7A1*, and *GRAMD3* genes.

The centromeric breakpoint closest to *LMNB1* was found in sample A14, and it was localized 9.8 kb upstream of the first exon of the *LMNB1* gene. The closest telomeric breakpoint to *LMNB1* was found in patient BR1, 1.8 kb downstream the last exon of *LMNB1*. The boundaries of the rearrangements in these two samples mark a ∼72 kb minimal critical duplicated region required for ADLD, between chr5:126,102,443 and chr5:126,174,517 and includes the *LMNB1* gene only (Fig. 1A). In addition to the duplication, families A6, A7, and K2–3 also showed the presence of a triplication of ∼13 kb, within the second intron of the *MARCH3* gene (Fig. 1, Supp. Fig. S1).

### Characterization of Tandem Duplication Junction Sequences

Duplication junction sequences were identified by long-range PCR by attempting to amplify across the unique duplication junction T1–C2, using outward facing primers and assuming a direct tandem orientation of the duplicated segment (Fig. 1B, Supp. Table S2). Healthy control samples were also used in the long-range PCR reactions to confirm that amplification only occurred from patient DNA (data not shown). Using this strategy, we were able to generate patient-specific amplification products from 15 duplication junctions in addition to the triplication junction. In families with multiple affected members, we confirmed that all affected individuals had identical duplications using the duplication junction-specific PCR primers.

**Figure 2:**
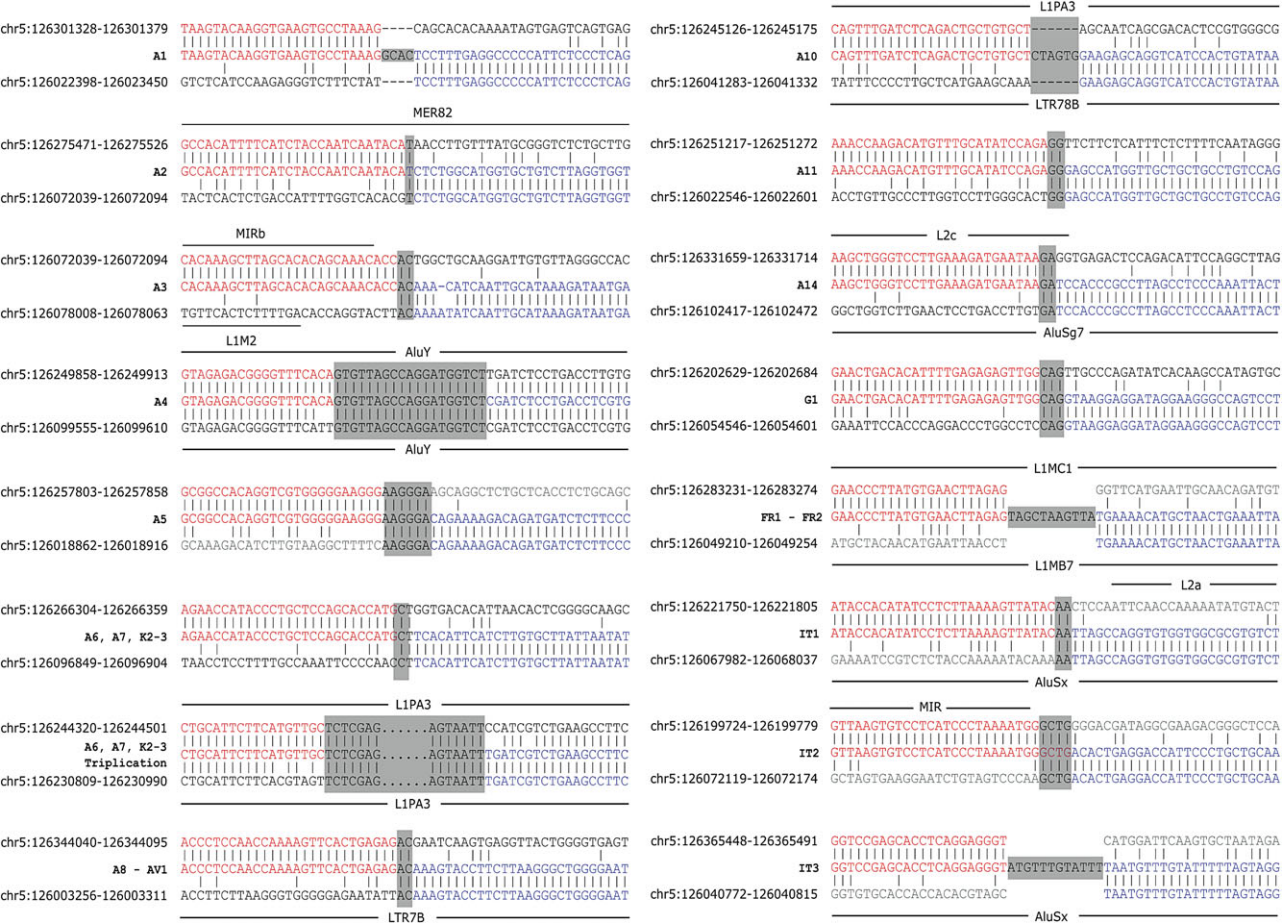
**LMNB*1* duplication junction sequences. Nucleotide positions from chromosome 5 (GCRh37/hg19) are indicated on the left of each junction. In each case, the reference sequence corresponding to the telomeric end of the duplication (red), the junction fragment present in **LMNB*1* duplication carriers (red and blue) and the reference sequence corresponding to the centromeric end the duplication (blue) are shown. The grey highlighted sequences represent either the presence of microhomology or nucleotide insertions at the duplication junctions. In sample A3, a single base pair deletion and an adjacent mismatch compared with the reference sequence were present. Repetitive elements present at the duplication junctions are also displayed. At the triplication junction (A6, A7, K2–3) the dotted line represents the extended part of the 146 bp segments that shows perfect homology.

Sequencing these PCR products allowed us to resolve all the 15 duplication junctions at the nucleotide level (Fig. 2 and Table 1). However, this technique did not allow identifying the breakpoints of the duplication in family BR1 (Fig. 1), where the junction was found to be more complex (described in detail in the next section).

Eleven of these 15 junctions showed short stretches of microhomology/overlap ranging from 1 to 6 nucleotides (Fig. 2 and Table 1). Four of the junction sequences (families A1, IT3, A10, FR1/FR2) showed the presence of an insertion of 4, 11, or 12 nucleotides. Interestingly, these duplications with insertions at their junctions also showed a clustering of their centromeric breakpoints within ∼25 kb (IT3, A10, and FR1/FR2 clustered within ∼8 kb) (Fig. 1A). Assuming a random distribution of breakpoints, this clustering was found to be statistically significant (*P* = 7 × 10^−3^).

Patient A3, had an insertion of one nucleotide and a deletion of the adjacent nucleotide, 3 bp from the duplication junction (Fig. 2). All other junction sequences matched perfectly with the reference sequence.

To identify the triplication junction in families A6, A7, and K2–3, we assumed that this was the result of a head to tail tandem duplication on one of the duplicated alleles (Fig. 1C). Using primers spanning this putative junction, we were able to obtain a PCR amplification product in the patient sample only, confirming the initial hypothesis. The ends of the triplicated segment showed a 146 bp homology and were found to lie in two directly oriented LIPA3 LINE elements of ∼6 kb in size that shared 96% sequence identity (Fig. 2).

### Characterization of an Inverted Duplication

**Figure 3:**
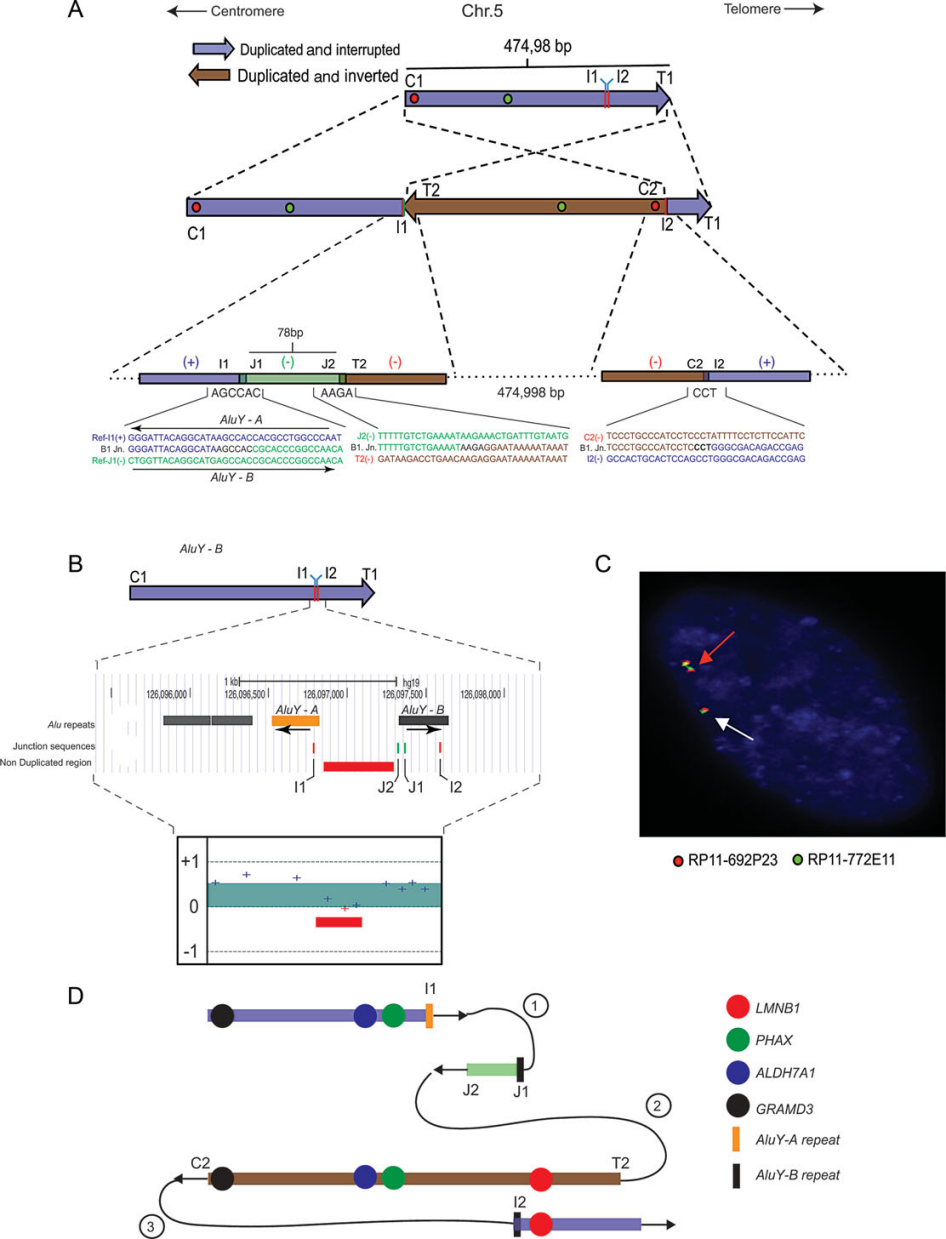
Architecture of the BR1 inverted duplication. A: Schematic representation of the inverted duplication in BR1. The C1 and T1 junctions represent the extents of the duplication. The duplicated segment C2–T2 (brown) is inverted and embedded between junctions I1 and I2 (red vertical lines). Analysis of the junction sequences reveals that the I1–T2 junction is complex with a 78 bp J1–J2 segment (green) interspersed within it. The J1–J2 and the T2–C2 segments are in the reverse orientation. The red and green circles mark the location of the BAC probes used for FISH. Sequence alignments of the I1–J1, J2–T2, and C2–I2 junctions (center) are shown with their respective reference sequences (above and below). In the sequence alignments the regions of microhomology are marked in black. The I1–J2 sequences fall within adjacent *AluY* repeats which are in an opposite orientations (arrows). B: Overview of the genomic region containing breakpoints I1–I2 (red vertical lines) and J1–J2 (green vertical lines) on the reference genome. Arrows mark the orientation of the *Alu* elements. The array CGH plot below shows the location of a nonduplicated segment (solid red horizontal bar) surrounded by a duplicated region in the BR1 sample. The *y*-axis represents relative probe intensity values on a Log_2_ scale. C: FISH analysis using the fluorescent labeled BAC probes RP11–692P23 (red) and RP11–772E11 (green). The red arrow points to the chromosome with the duplicated allele, whereas the white arrow shows the chromosome with the normal allele. The presence of a red–green–green–red pattern confirms the presence of the inverted duplication. The normal chromosome shows a red–green pattern. D: Model showing the replication fork switching that could give rise to the BR1 duplication. Relative locations of the duplication junctions are marked together with the *Alu* repetitive elements and genes involved in the rearrangement. Arrowheads show direction of DNA relative to the positive strand. Circled numbers represent FoSTeS events. Colored circles represent the duplicated genes.

In the duplication in family BR1, the strategy of using outward primers to amplify across duplication junctions did not yield a product. Using the junction coordinates determined by aCGH, we used inverse PCR to identify the sequences flanking the centromeric and telomeric duplication breakpoints. This revealed the presence of complex duplication junction architectures (Fig. 3A). The C2 breakpoint corresponded to position chr5:125,699,519 on the reference genome (all coordinates are for chr. 5). This was flanked by a segment in the opposite orientation that began at position chr5:126,097,581 (breakpoint I2, Fig. 3A). Breakpoint I2 was located within an *Alu* repeat, and the transition was marked by a “CCT” microhomology sequence (Fig. 3A).

The T2 breakpoint corresponded to position 126,174,517 and it was flanked by a sequence that began at position 126,097,260 in direct orientation (J2, Fig. 3A). The sequence transition was marked by a “AGAA” microhomology. This segment continued for 78 bp into the breakpoint J1, and then transitioned to a different segment beginning at position 126,096,808 (junction I1) in the opposite orientation. This junction was marked by a microhomology of six base pairs (AGCCAC). The J1 and I1 breakpoints were located within *Alu Y* repeats adjacent to each other (*AluY-*A and *AluY-*B) with a sequence identity of 89% but in opposite orientations. The J1 breakpoint was in the same *AluY-*B repeat as the I2 junction (Fig. 3B).

This sequence configuration suggested that the entire duplicated segment (C2–T2) corresponding to ∼475 kb had been inverted and embedded between the breakpoints I1 and I2 (Fig. 3A). PCR primers designed to amplify across these duplication junctions were able yield a product in the patient sample but not in control samples from unaffected individuals. Previous reports have described tandem duplications that have been linked by sequence fragments that are in an inverted orientation leading to a duplication-inverted triplication–duplication structure [Carvalho et al., 2011]. To determine if this was the case in our patient we reexamined the aCGH plot in vicinity of the insertion sites, that is, between I1and I2 (Fig. 3B) but we did not observe probes signals with a log ratio corresponding to a triplication. On the contrary, three probes with signals corresponding to a copy number of one were surrounded by probes with signals corresponding to a duplication (Fig. 3B). This suggested that during the formation of the duplication, there has been a loss of ∼500 bp from the interrupted segment (Fig. 3B).

Fluorescence in situ hybridization analysis (FISH) with probes mapping on the middle and the end of the duplicated segment confirmed the presence of the inverted duplication (Fig. 3C). The given hybridization pattern was observed in more than 70% of enlarged interphase nuclei. In the remaining cells, sufficient resolution could not be achieved to allow us to identify any discernable structure.

### Analysis of Genomic Architecture of Duplication Breakpoints

We analyzed the genomic architecture of the centromeric and telomeric breakpoints in the 16 independent duplications to determine if they played a role in the rearrangement process. The triplication junction was not included in these analyses, as it seemed to be the result of an event independent from the *LMNB1* duplication.

We did not identify any LCR (also known as segmental duplications) in the genomic region surrounding the *LMNB1* gene within 100 kb of any of the duplication breakpoints.

Thirteen of the 32 analyzed breakpoints (41%) were within repetitive sequences, as defined by the Repeat Masker software (Fig. 2, Table 1). In four patients, both the proximal and distal junctions were located in repetitive elements. Among these, the duplication in family A4 had two *Alu*Y repeats sharing ∼90% identity at both ends. In the remaining patients, no significant sequence identity was found between the centromeric and telomeric breakpoints.

**Figure 4:**
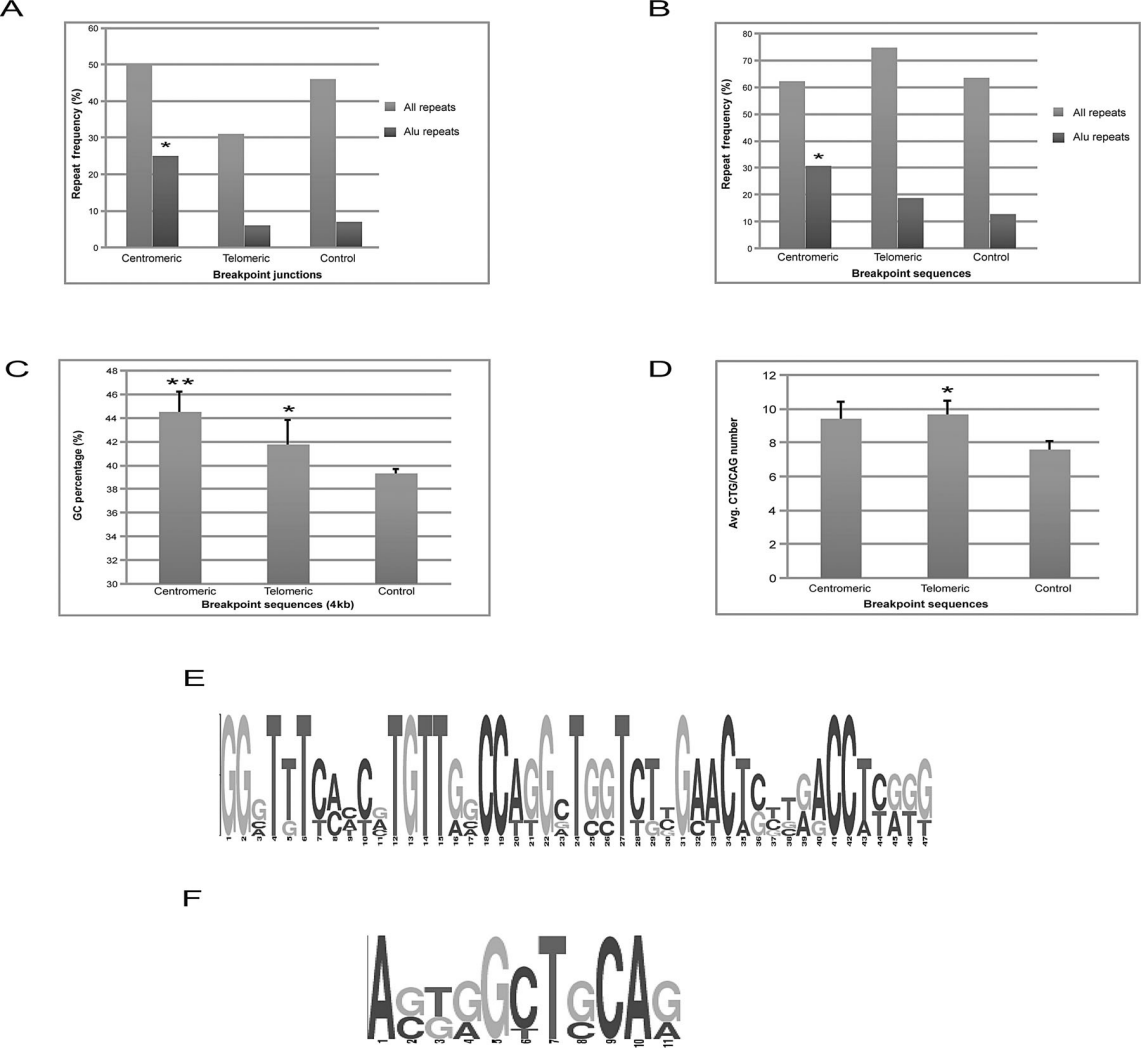
Bioinformatics analysis of duplication breakpoints and surrounding genomic regions. A: Analysis of repetitive elements at duplication junctions. Light gray columns show all repetitive elements and dark gray columns show *Alu* repetitive elements only. An enrichment of *Alu* repetitive elements in centromeric breakpoints was present. B: Analysis of repetitive elements in 200 bp sequences surrounding duplication breakpoints in patients versus simulated breakpoints of control sequences. Only centromeric sequences show an enrichment of *Alu* repetitive elements. C: GC% in 4 kb sequences surrounding duplication breakpoints in patients versus simulated breakpoints in controls. All breakpoint sequences show significantly higher GC% than control sequences. D: Enrichment of CTG/CAG motifs in duplication breakpoint sequences. CTG/CAG motifs were found to be significantly enriched in telomeric breakpoint sequences. In all panels asterisks (*) represents a statistical significance of *P* < 0.05, and double asterisks (**) represents *P* < 0.001. E: Consensus sequence motif at centromeric duplication breakpoints. F: Consensus sequence motif at telomeric duplication breakpoints. In both (E) and (F), *x*-axis represents position of the nucleotide in the motif and the height of the nucleotide represents the probability of observing that particular nucleotide at that position. Both motifs were found to significantly overrepresented in the respective patient breakpoint sequences when compared with control sequences (*P* < 10^−6^).

We did not find a significant enrichment of repetitive elements at these breakpoints compared with 500 randomly selected control sequences. However, considering *Alu repetitive elements* alone, we found that four of the 16 (25%) centromeric breakpoint sequences were within an *Alu* element (Fig. 4A) compared with 34 of 500 (7%) control sequence breakpoints (*P* = 0.02, Fisher’s exact test). A similar result was observed when the 200-bp region surrounding the breakpoints was compared with the control sequences (Fig. 4B). No such difference was observed when telomeric breakpoints were analyzed (Fig. 4B).

As an increased GC% has been associated with greater instability of duplications in the region of *MECP2* [Bauters et al., 2008], we sought to determine the GC content of the *LMNB1* duplication breakpoints. The GC contents of the centromeric (44.5%) and telomeric (41.8%) breakpoint sequences were significantly higher when compared with control sequences (39.3%) (*P* = 1.6 × 10^−8^ for centromeric and *P* = 0.005 for telomeric sequences, Student’s *t*-test) (Fig. 4C).

Given the difference in the *Alu* repeat enrichment and GC content between the centromeric and telomeric breakpoints, we investigated whether this was the result of differences in the overall composition of the genomic regions in which these breakpoints were located. We arbitrarily chose a ∼600 kb region centered on the *LMNB1* gene. The centromeric half of this region revealed a much higher *Alu* density (37.1%) compared with the telomeric half (9.1%) or the whole chromosome 5 (8.4%) (Supp. Table S3). Analysis of the GC content of this 600 kb region found that it was 42% (43.1% for the centromeric half; 40.1% for the telomeric half) compared with 39.2% for the whole chromosome 5 (Supp. Table S3).

### Analysis of Sequence Motifs at Breakpoints

None of the 40 sequence motifs previously reported to predispose to DNA breakage [Vissers et al., 2009] were found to be statistically overrepresented at patients’ breakpoints (Supp. Table S4).

Previous reports have suggested that the trinucleotide sequence CTG/CAG is enriched in the vicinity of *MECP2* and *PLP1* duplication junction sequences [Carvalho et al., 2009]. We found an increased frequency of the CTG/CAG motif at the telomeric breakpoints when compared with control sequences (*P* = 0.02, Student’s *t*-test). We did not observe an enrichment of CCG/GGC motifs (data not shown) suggesting that the enrichment of the CTG/CAG motif was unlikely to be simply a result of differences in GC content between patient and control sequences.

Using the MEME software, we searched for the presence of novel motifs in the duplication breakpoint sequences. At centromeric breakpoints we found the ‘GGVTKTYMHYVTGTTRVCCWKGVTSSTYKBGMWCWSBBRRCCWYRKK’ motif significantly enriched (five of the 16 breakpoints, *P* = 8.6 × 10^−8^, Fisher’s exact test) (Fig. 4E). On further examination, we determined that this motif was part of *Alu* elements in four of the five breakpoint sequences suggesting that the *Alu* elements were responsible for the motif. At telomeric breakpoints the motif “ASKRGCTSCAR” was significantly overrepresented (six of the 16 breakpoints, *P* = 8.5 × 10^−9^, Fisher’s exact test) (Fig. 4F). We were unable to determine whether this sequence motif represented a known structural or functional DNA element.

We found non B-DNA conformations (Z-DNA, cruciform, and triplexes), known to be implicated in DNA rearrangements, only at the telomeric breakpoint sequence of patient A10 (Z-DNA forming sequence “CCGTACGTGTGCACAGGGGCATGG”).

### Chromosomal Origin and Haplotype Analysis of Duplications

To determine whether the duplications were the result of inter- or intrachromosomal rearrangements, we typed eight microsatellite markers across the duplicated segment (Fig. 1A and Table 2). We did not find triple alleles in any of the samples strongly suggesting that the duplications resulted from intrachromosomal rearrangements.

**Table 2:** Haplotype Analysis of ADLD Patients

S. No	Family ID	Microsatellite marker alleles (bp)							
		Q1	Q2	Q4	Q5	Q6	Q7	Q10	Q8
1	A1	337	211	199	480	186	248	238	317
2	A2	337/345	221	191	480	200	250	266	311
3	A3	345	263	191/193	492/506	194	248/250	238	315
4	A4	343	223	195	483	200	250	266	311
5	A6, A7, K2–3	349	227	193	492	172	248	238	317
6	A8, AV1	347	253	193	492	198	250/252	238	315
7	A11	347	255	197	477	202	250	264/278	311
8	FR1, FR2	353	237	193	498	200	248	238	317
9	IT1	345	253	191/193	492	200	254	266	313
10	IT3	335	203	195/197	492	184/194	256	238/266	315

Microsatellite markers are arranged according to their order along chr. 5 from centromere to telomere. Shaded boxes represent the extent of the duplications in different patients. Numbers in each box represent alleles as fragment sizes in base pairs (bp). For some families we could not determine the phase at all loci. In these cases, both alleles are shown.

As described above, three of the duplications were shared by more than one family (Fig. 1A, Table 1). In these cases, families with identical duplications had the same haplotype on the duplicated allele suggesting that they arose from the same mutational event derived from a common founder. In 10 of the 16 independent duplications, we had two or more affected members and were able to phase the alleles on the duplicated segment and compare haplotypes associated with the duplications. We did not observe haplotypes shared among families with different duplication sizes suggesting that an “at risk” chromosomal haplotype is unlikely (Table 2).

### Expression Analysis of LMNB1 at mRNA and Protein Levels

**Figure 5:**
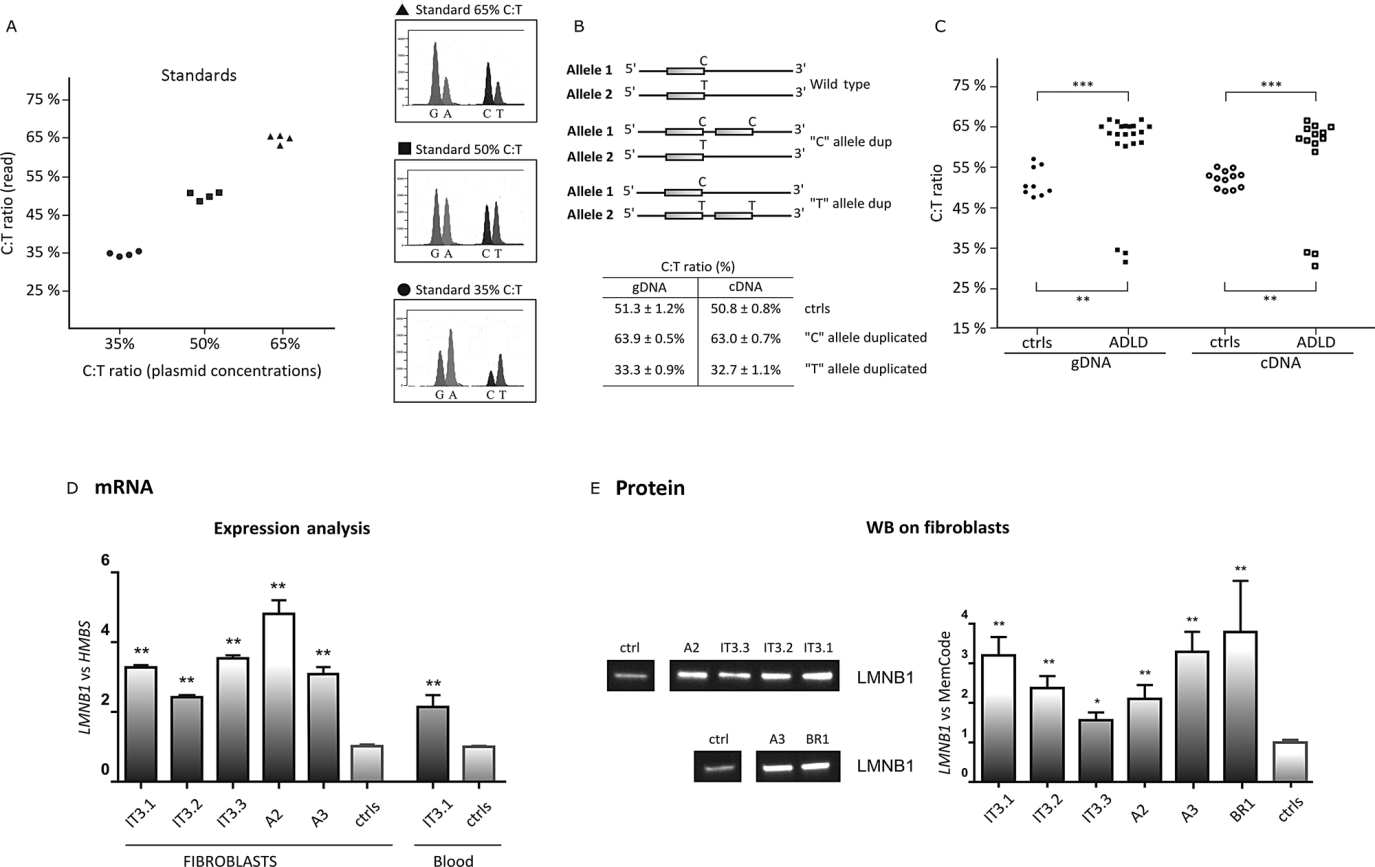
Lamin B1 expression analysis. A: Calibration of the SNaPshot experiment using known concentrations of two plasmids containing the C and T allele of SNP #rs1051644. Percentages indicate the C:T ratio. A reproducible correlation between expected (*x*-axis) and measured (*y*-axis) values were obtained. On the right, electropherograms at different relative concentrations. B: Scheme of the wild-type heterozygous SNP rs#1051644 and of the two possible duplication configurations. The table on the bottom shows the results of the SNaPshot experiments whose graphic is in panel C (values = mean ± standard error). C: SNaPshot results of the rs#1051644 analysis on genomic DNA (gDNA) and cDNA derived from fibroblasts of controls (ctrls) and patients (ADLD), showing the C:T ratio (*y*-axis). Controls are shown as black-filled circles (gDNA) and empty circles (cDNA), patients are shown as black-filled squares (gDNA) and empty squares (cDNA). Heterozygous controls cluster around 50%, whereas duplication carriers cluster around 65% or 35% depending on which of the two alleles is duplicated (***P < 0.001; **P < 0.01). D: Real-time experiments measuring **LMNB*1* cDNA levels compared with the reference gene *HMBS*. Patients showed a statistically significant increase compared with controls both on mRNA derived from fibroblasts and from blood (**P < 0.01). E: Western blot analysis shows increased LMNB1 expression in patients compared with control samples (samples were normalized using the MemCode system); full Western blot images and MemCode staining are available in Supp. Fig. S1). On the right, the OD quantification of LMNB1 compared with MemCode staining. In all patients, LMNB1 protein levels were significantly increased compared with controls (**P < 0.01; *P < 0.05).

To determine the relative contribution of the normal and duplicated *LMNB1* alleles to gene expression, we set up a primer extension assay exploiting a polymorphism in the 3′-UTR of the lamin B1 gene (rs#1051644): six patients (IT1, IT2, IT3, FR1, FR2, and US1) were heterozygous and could be used in this assay. Based on the calibration curve, all patients carrying a duplication showed a genomic DNA ratio between the two alleles of the rs#1051644 SNP of 65%–35%. A similar ratio was seen when the assay was carried out on cDNA from patient fibroblasts and blood (Fig. 5A–C). This indicated that the three lamin B1 gene copies in a duplication carrier were equally expressed.

Real-time PCR on cDNA derived from fibroblasts or blood showed an increase in *LMNB1* expression ranging from 2.1 to 4.8 relative to the control samples, whereas expression was found to range from 1.6 to 3.2-folds at the protein level (Fig. 5D–F). Expression levels in patients were significantly higher than controls both at the RNA and protein levels. Variability in expression levels was also noted among members of the same family (Fig. 5, subjects IT3.1, 3.2, 3.3). This suggested that it was unlikely that expression in patient samples was correlated with the size of the duplication and that differences in expression maybe due to experimental variations or differences in cell culture conditions.

## Discussion

We studied a group of twenty ADLD families by high-resolution aCGH to map the *LMNB1* duplication boundaries. Two cases allowed us to define the minimum critical duplicated region required for the development of ADLD which was ∼72 kb, and extended from ∼9.9 kb upstream of the 5′-UTR, and ∼1.8 kb downstream of the 3′-UTR of *LMNB1* (Fig. 1A). *LMNB1* is the only gene contained in this region and no other gene is even partially duplicated. This confirms that the duplication of *LMNB1* alone is sufficient to cause ADLD.

Clinical features of ADLD patients were similar in all patients in whom *LMNB1* was the only gene completely duplicated, and we did not notice differences associated with different duplication extents. The only possible exception is patient BR1, in whom the initial symptoms were not autonomic dysfunction. The BR1 duplication was the only one that encompassed the complete coding regions of other genes. This large inverted duplication involved three genes centromeric to *LMNB1*, namely *GRAMD3*, *ALDH7A1* (MIM #107323), *PHAX* (MIM #604924). This may suggest that the involvement of one of these genes may play a role as modifier of the disease phenotype or that the clinical spectrum of ADLD, particularly concerning the symptoms at onset, is wider than expected.

Genotyping microsatellite markers around the *LMNB1* gene in patients revealed two important characteristics regarding the ADLD duplications: (1) subjects with identical junctions shared the same haplotype, suggesting the presence of a common founder. It confirms that *LMNB1* duplications are indeed nonrecurrent and that identical duplications in different families derived from the same mutational event; (2) the duplications were the result of intrachromosomal rearrangements. This is similar to *MECP2* and *PLP1* duplications that were also shown to arise from intrachromosomal events [Bauters et al., 2008; Inoue et al., 1999].

Fifteen of the 16 duplications (94%) had a “simple” head to tail tandem orientation as defined by the fact that there was only a single duplication junction. One of these also showed a triplication (families A6, A7, K2–3). It is likely that the triplication arose subsequent to the original *LMNB1* duplication through an independent repeat-mediated NAHR mechanism mediated by the flanking LIPA3 LINE repeats. It thus represents a second duplication event on one of the duplicated alleles and we have counted this event as a simple duplication. The only “complex” duplication was found in patient BR1, and consisted of an inverted duplicated segment. Compared with other diseases in which nonrecurrent duplications have been analyzed in detail such as PMD, developmental delay caused by *MECP2* duplications and Potocki-Lupski microduplication syndrome (PTLS), the percentage of complex duplications in ADLD appears to be much lower [Carvalho et al., 2009; Lee et al., 2007; Zhang et al., 2009a; 2009b]. It is unclear if this over representation of simple duplication events is a characteristic of the mechanisms involved in the ADLD duplications.

The identification, for the first time, of a large number of *LMNB1* duplication junction sequences has allowed us to speculate on the mechanisms that may underlie these genomic rearrangements. Given that LCRs do not flank the *LMNB1* gene and the duplications are nonrecurrent, nonallelic homologous recombination (NAHR) is an unlikely mechanism for the generation of most of the *LMNB1* duplications, with the exception of the *Alu–Alu*-mediated rearrangements in patient A4 and the triplication in families A6, A7, K2–3. *Alu–Alu*-mediated duplications have been reported throughout the genome and *Alu* elements with identities as low as 76% have been shown to mediate tandem duplications [O’Neil et al., 2007]. However, recent reports have also suggested that replication mechanism such as FoSTeS/MMBIR can also explain *Alu–Alu*-mediated rearrangements [Vissers et al., 2009; Zhang et al., 2009a]. The lack of LCRs around *LMNB1* is also interesting. This is in contrast to other well studied diseases caused by nonrecurrent duplications, such as those involving the *PLP1*, *MECP2* genes and PTLS, where LCRs are thought to play an important role in duplication formation [Becker et al., 2011; Carvalho et al., 2009; Lee et al., 2006; Woodward et al., 2005; Zhang et al., 2009b].

Four of the 16 patients showed an insertion at the duplication junctions ranging from four to 12 nucleotides. Insertions are usually a hallmark of NHEJ mechanisms and represent “information scars” at the repair sites of double stranded breaks (DSB) [Lieber, 2008; McVey and Lee, 2008]. It is interesting that the centromeric breakpoints of these four patients clustered within 25 kb of each other, a grouping that was statistically significant. This might indicate that these duplications share a common mechanism mediated by the genomic architecture surrounding their centromeric breakpoints.

The majority of the duplication junction sequences (11 of 16) show the presence of an overlap of between 1 and 6 bp with 2 bp being the most frequently observed microhomology. Microhomology at duplication and deletion junctions has been a defining feature in numerous studies involving rearrangements associated with diseases such as PMD and *MECP2* associated developmental delay [Carvalho et al., 2009; Woodward et al., 2005]. It has been shown that 75% of tandem duplications and 80% of deletions associated with pathogenic CNVs contained regions of microhomolgy at their junctions [Vissers et al., 2009]. The presence of microhomology at rearrangements junctions is usually a signature of a nonhomologous repair process NHEJ or alternative NHEJ (also known as microhomology-mediated end joining (MMEJ). These NHEJ mechanisms have been implicated in both normal copy number variations and duplications and deletions associated with such disease [Carvalho et al., 2009; Vissers et al., 2009; White and den Dunnen, 2006; Woodward et al., 2005].

The genomic rearrangement in BR1 is a complex duplication difficult to explain using an NHEJ model, but compatible with a replication fork switching mechanism such as FoSTeS/MMBIR. Inverted *Alu* elements have been shown to predispose to replication fork stalling, double stranded breaks and inverted duplications [Lobachev et al., 2002; Voineagu et al., 2008]. While these repeats have been close enough to form cruciform structures, evidence has suggested that inverted repeats, even at a distance, can lead to inversions [Carvalho et al., 2011]. We propose a model whereby the presence of the inverted *Alu* element results in a replication blockage that causes a template-switching event in the vicinity of I1. We propose that three template-switching events occurred to produce the complex rearrangement (Fig. 3D): (1) the replication fork switches to the opposite sister chromatid because of the homology of an inverted *Alu*Y*-*B element (first junction sequence I1–J1, Fig. 3). (2) After progressing for a short distance (78 bp), the nascent strand disengages and a microhomology-mediated migration to another replication fork occurs. This is not mediated by an inverted repeat and thus maintains the inverted orientation (second junction sequence J2–T2). (3) Replication progresses for ∼475 kb, resulting in the duplication of the *LMNB1*, *PHAX*, *ALDH7A1*, and *GRAMD3* genes, and finally a third template-switching event causes the migration of the replication fork back to the strand in the direct orientation, again mediated by a microhomology (third junction C2–I2). This complex event results in the final configuration of the inverted duplication observed in BR1.

An analysis of the genomic architecture surrounding the breakpoint junctions suggests a number of features that may predispose the region to genomic instability leading to the *LMNB1* duplication; the most striking of which is the involvement of *Alu* repetitive elements at the duplication breakpoints. One of the duplications also shows the presence of *Alu elements* at both ends. In addition, they are also involved in the insertion junctions of the complex BR1 duplication. The enrichment of *Alu* repetitive elements is most striking around the centromeric duplication junctions and this increased frequency is likely a consequence of an enrichment of *Alu* sequences in the centromeric part of the genomic region surrounding the *LMNB1* gene. *Alu* repeat enrichment has been previously reported for *MECP2* duplication junctions and in the vicinity of LCRs [Bailey et al., 2003; Bauters et al., 2008]. A higher *Alu* density in the *MSH2* gene was shown to be associated with an increased frequency of *Alu*-mediated deletions [Li et al., 2006]. Thus, although there is a clear association between *Alu* elements and genomic rearrangements, the exact mechanisms are unclear. ADLD duplication junctions, in particular centromeric boundaries, also showed a higher GC%. *Alu*-mediated deletions have been shown to occur in regions with a high GC content (∼45%) [Sen et al., 2006] and a high GC content was associated with early replicating regions as well as an increased frequency of DNA breaks in neuroblastoma translocations [Stallings, 2007].

We also noted an increased frequency of CTG/CAG trinucleotides at the telomeric duplication breakpoints. Their enrichment was originally found in *E. coli*, at junctions produced by gene amplification induced under stress conditions [Slack et al., 2006], and they have also been found near *MECP2* and *PLP1* duplication breakpoints [Carvalho et al., 2009]. It has been suggested that the CTG/CAG motifs may represent a relationship between the ends of Okazaki fragments and the involvement of the lagging strand in a long distance template-switching model [Slack et al., 2006]. It is interesting that this motif is significantly enriched in the telomeric breakpoint sequences as these may represent sites of template switching between the replication forks.

Which mechanism is likely to be responsible for the ADLD duplications? Given that the molecular signatures of both NHEJ/MMEJ and replication-based mechanisms such as FoSTeS/MMBIR overlap, it is difficult to answer that question definitively. It is also possible that more than one mechanism is at play. However, several of lines of evidence favor a replication-based FoSTeS/MMBIR mechanism. Studies have shown that human fibroblast subjected to replication stress can result in a high frequency of novel CNVs also characterized by short stretches (<6 bp) of microhomology at their junctions thus suggesting a mitotic origin for CNV formation [Arlt et al., 2012]. FoSTeS/MMBIR occurs during mitosis, whereas NHEJ appears to be downregulated during mammalian meiosis [Fiorenza et al., 2001]. In addition to explaining simple tandem duplications FoSTeS/MMBIR mechanisms can also more readily explain the presence of complex rearrangements and the incorporation of stretches of DNA from multiple different genomic locations such as that observed in the case of the BR1 duplication [Zhang et al., 2009a].

In patients’ fibroblasts, the expression analysis confirmed an increase of *LMNB1* both at mRNA and protein levels. We demonstrated that the duplicated and normal *LMNB1* alleles in ADLD patients show equal expression, suggesting regulatory regions are maintained within the rearranged segment. Given the presence of three *LMNB1* alleles, the theoretical increase of its expression is 1.5-fold. We demonstrated that the differences between expected and observed values for *LMNB1* expression are not due to the duplicated allele alone, because the three *LMNB1* alleles were always equally expressed. This may suggest that in case of duplication, the *LMNB1* mRNA/protein accumulates in patients’ cells. Such expression increments and the variability among patients were also found for mRNA and protein levels in nerve biopsies from patients with duplications of the *PMP22* gene. Its origin was unknown, and it did not correlate with disease severity [Katona et al., 2009].

In conclusion, we have carried out an analysis of the largest collection of ADLD families caused by *LMNB1* duplications, to date. We have been able to identify and analyze all the duplication junctions at the base pair level. In contrast to previous reports, we show that *LMNB1* duplications can have a heterogeneous architecture with the first description of an inversion involving *LMNB1*. We propose that the genomic architecture, including the enrichment of *Alu* repetitive elements and higher GC%, especially in the genomic region centromeric to the *LMNB1* gene may play an important role in mediating the ADLD duplications. Given the overlapping signatures of the different duplication generating mechanisms it is difficult to identify unambiguously which of these is functioning in ADLD. It is also possible that there may be more that one mechanism at play. Our results suggest that NHEJ/MMEJ and replication-based mechanisms such as FoSTeS are likely to play an important role in the formation of the duplications that cause ADLD.

## Notes

Disclaimer: Supplementary materials have been peer-reviewed but not copyedited.
