# Supplementary material for: Analysis of LMNB1 Duplications in Autosomal Dominant Leukodystrophy Provides Insights into Duplication Mechanisms and Allele-Specific Expression
Source: Hum Mutat. 2013 May 28;34(8):1160–71. doi: 10.1002/humu.22348 (PMC3714349; doi:10.1002/humu.22348)
Supplement: Supplementary file 1 [file humu0034-1160-sd1.pdf]

Supp. Materials and Methods

Genotyping analysis

Eight microsatellite markers surrounding the *LMNB1* gene were identified using the 'Microsatellite' track on the UCSC genome browser (Supp. Table S5). Microsatellite PCR products were fluorescently labeled using a 6-FAM-fluorophore using a protocol described previously [Schuelke, 2000]. Fluorescently labeled PCR products were run on an ABI-Prism 3730 automated DNA sequencer (Applied Biosystems, Foster City, CA, USA). Results were analyzed using the PeakScanner software (Applied Biosystems, Foster City, CA, USA).

Expression analysis of the LMNB1 Gene

Total RNA was extracted from PAXgene samples using the PAXgene Blood RNA Extraction Kit (Qiagen) as described by the manufacturer, and from fibroblast using the miRNeasy Mini Kit (Qiagen). RNA concentration was measured by spectrometry (NanoDrop 1000, Thermo Scientific, Wilmington, DE, USA) and cDNA was generated using the Transcriptor Reverse Transcriptase kit (Roche, Mannheim, Germany) from 1µg of total RNA.

The expression levels of *LMNB1* and the reference gene hydroxymethylbilane synthase (*HMBS*) were measured with predesigned TaqMan assays (Applied Biosystems, *LMNB1*, Hs01059210_m1; *HMBS*, Hs00609297_m1).

Reactions were carried out in triplicate on an ABI 7500 real-time PCR machine using the ABI 2X TaqMan Gene Expression master mix or TaqMan Universal PCR Master Mix II, according to the manufacturer's instructions (Applied Biosystems, Foster City, CA, USA). At least three healthy controls were added in each experiment.

Immunoblot analysis

Total proteins were extracted from fibroblasts in RIPA buffer. Forty µg of protein extracts were run on 4-15% mini-PROTEAN TGX gels, then blotted onto nitro-cellulose (BIO-RAD, Hercules, CA, USA) in Tris/Glycine buffer with 20% methanol at 4°C for an hour. Protein transfer efficiency was evaluated using the MemCode Reversible Protein Stain Kit (Pierce Biotechnology Rockford, IL, USA) (Supp. Fig. S2). Lamin B1 was detected using primary anti-lamin B1antibodies (Abcam) and WesternBreeze™ Chemiluminescent Detection Kit

(Invitrogen, Carlsbad, CA, USA). Images were captured with a ChemiDoc™ XRS+ System and densitometric analysis was performed with Image Lab™ Software (Bio Rad, Milan, Italy).

FISH analysis

Dual color fluorescence in situ hybridization (FISH) analysis was performed on interphase nuclei from fibroblast cultures from the patient from the BR1family. Two clones RP11-692P23 and RP11-772E11 (AC135791) located within the 5q23 duplication region were labeled directly with Spectrum Orange-dUTP or Spectrum Green-dUTP (Abbot Molecular/Vysis, Des Plaines, IL, USA) following hybridization according to the manufacturer's protocol. Fifty interphase cells were scored and digital images were captured by a Power Macintosh G3 System and MacProbe version 4.4 (Applied Imaging; San Jose, CA, USA). Enlarged nuclei were specifically analyzed to achieve higher resolution and resolve the duplication structure.

Supp. References

Schuelke M. 2000. An economic method for the fluorescent labeling of PCR fragments. Nat Biotechnol 18(2):233-4.

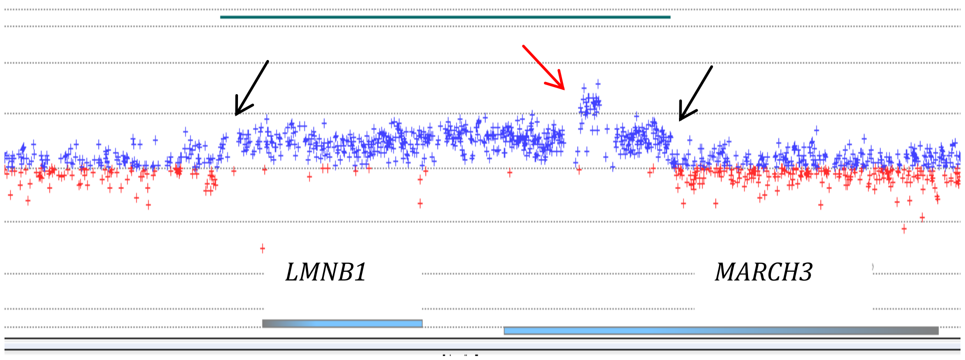

Supp. Figure S1. Representative array CGH plot. Array CGH plot from patient A7 showing from the region of chr. 5 surrounding the *LMNB1* gene. The boundaries of the duplication are marked by black arrows. The red arrow shows the triplication within the duplication. The genomic extents of the *LMNB1* and *MARCH3* genes are shown below.

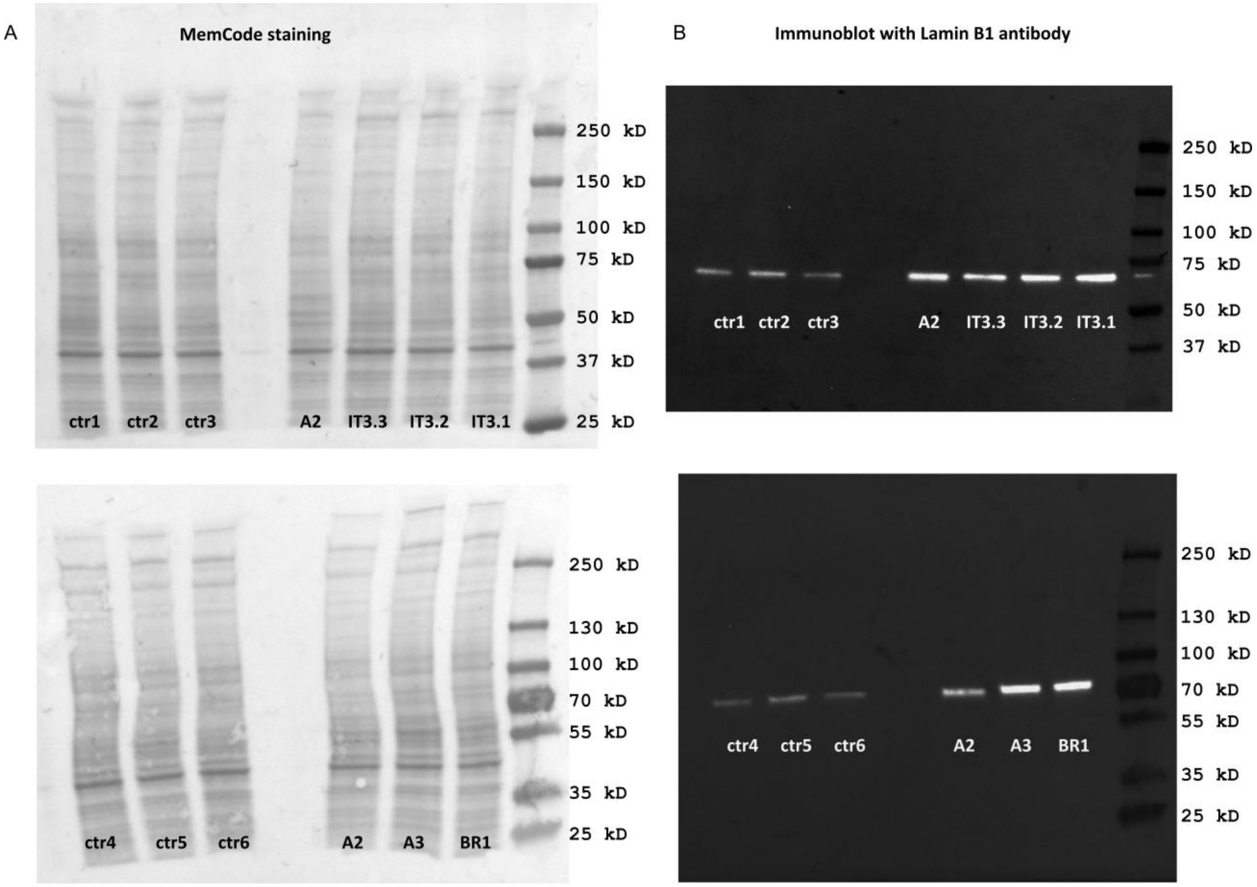

Supp. Figure S2. Full MemCode and Western blot gels staining. A) Two images showing the MemCode staining (Pierce Biotechnology) used to normalize protein quantities. On the top, the marker used is the “Precision Plus Protein Standard” (Biorad) and, on the bottom, the “PageRuler Plus” (Biorad). The weight of each band is reported on the right. B) The same two gels of panel A are shown after staining with anti-LMNB1 antibody. The markers are the same as in panel.

Supp. Table S1. ADLD patient details

S.No.	Family ID	No. of affected samples	Published previously	Country of origin
1	A1	2	Meijer et al., 2008	Canada
2	A2	2	Schuster et al., 2011	Sweden
3	A3	2	Schuster et al., 2011	Sweden
4	A4	3	Fogel et al., 2012	India
5	A5	1	Unpublished	USA
6	A6	1	Unpublished	USA
7	A7	1	Unpublished	USA
8	K2-3	1	Unpublished	USA
9	A8	1	Unpublished	USA
10	AV1	1	Unpublished	USA
11	A10	1	Schuster et al., 2011	Israel
12	A11	2	Schuster et al., 2011	Germany
13	A14	2	Unpublished	USA
14	GR1	2	Dos Santos et al., 2012	Germany
15	FR1	2	Unpublished	France
16	FR2	1	Unpublished	France
17	IT1	2	Brussino et al., 2009	Italy
18	IT2	1	Unpublished	Italy
19	IT3	2	Guaraldi et al., 2011	Italy
20	BR1	1	Unpublished	Brazil

Supp. Table S2. Primers and annealing temperature used for breakpoints identification

S.No.	Family	PRIMERS (Forward/Reverse)	Ta (°C)
1	IT1	5'CAAATTGTCAAGTTAATTGCTTTTAGCC 3'<br>5'ATAATGGCAGAAATAAAATCCTCAGTGC 3'	57
2	IT2	5'AAAGTGAAATACTCCTGGAGAACAAGACC 3'<br>5'TTCTTGGAAAGCAAGTAGACTGAAATAAG 3'	57
3	IT3	5'AAAAACAAGCTGCTTTTACCCCCTTAC 3'<br>5'TACATTATATCCATTCACATGAACAAAGC 3'	57
4	FR1, FR2	5'GTGAAACTGACTTTTAGTTTGGTGTATCC 3'<br>5'GTATGAGAATCCAAATTCCTCTACAGC 3'	57
5	US1	5'GTTCTAATCTCAGAAGAGTGGATCTAACC 3'<br>5'TACATTATATCCATTCACATGAACAAAGC 3'	57
6	A1	5' AGGCTTCCATTTCTTGAGCA 3'<br>5' ATAATAAGGCCGGGCACAGT 3'	58
7	A2	5' GAGGGTTGACAAAAGGTCCAG 3'<br>5' TCACGAGATCAAGAGATCCAGA 3'	58
8	A3	5' ATTGGGTGGGAATAGTCAACTG 3'<br>5' AGTGGTCACAGTATGCCATGAG 3'	58
9	A4	5' GGGGCAAGTCAAGTAAGTTTTG 3'<br>5' GGAATCACTTCAGTTCAGGACA 3'	58
10	A5	5' AGGTTGCCACTGGTAAGTGATT 3'<br>5' AACACTAGGACCCACTCCGATA 3'	58
11	A6, A7, K2-3	5' AGCAAGTCAAGGGCATCATC 3'<br>5' CCCGGCCAACAATTCTTTA 3'	58
12*	A6,A7,K2-3	5' CTTACTCACTAGGCTGCGTTCA 3'<br>5' AGGGGTCACAGATTTTCTGAAG 3'	58
13	A8, AV1	5' CTTCCCATACCCCAGTGGTT 3'<br>5' CCTTGCGATAACGTACTTTGC 3'	58
14	A10	5' CACGCAGAAGTGGAGCCTAT 3'<br>5' ATTAGCCTTTGGGGGAGTAAAG 3'	58
15	A11	5' TTCTGGGCACTCTAGAGCACTT 3'<br>5' CACGCCCAGCCTCTATTTTAT 3'	58
16	A14	5' CTAAGGGCAGGAAGAATGAGAA 3'<br>5' AAGTAGGGGAAGATGCATTCAG 3'	58
17	G1	5' GGTGGATCACAAGGTCAAGAGT 3'<br>5' GGACTCGAGCTGGTTGTAATCT 3'	58
18#	BR1-Inv-Centromere	5' TGCCACCAACTATTCAAGTGAC 3'<br>5' CCTCTTCACATCATGCTGCTT 3'	58
19#	BR1-Inv-Telomere	5' AGCTGCACCTAAACCCATTC 3'<br>5' CTCAGGAATCTACTACTGCCACAC 3'	58

*: Primers used for triplication junction. #: Primers used for inverse PCR

Supp. Table S3. Features of ~ 600Kb genomic region surrounding the *LMNB1* gene

	**Centromeric half**	**Telomeric half**	**Whole chr. 5**
**Coordinates**	125,840,514-126,142,513	126,142,514-126,444,513	1- 180,915,260
**GC%**	43.1	40.9	39.2
**Alu repeat density (%)**	37.1	9.1	8.4

Supp. Table S4. DNA breakage associated motifs in breakpoint sequences

Motif Name	Motif	No. of sequences with motif				
		Patient sequences		Control Sequences (n=500)	P Value	
		Cent. (n=16)	Telo. (n=16)		Cent.	Telo.
Nonamer recombination signal	ACAAAAACC	-	-	2	-	-
DNA polymerase a/b frameshift hotspot 1	ACCCCA	1	1	40	0.739	0.739
DNA polymerase b frameshift hotspot 1	ACCCWR	7	5	169	0.282	0.676
Translin-binding site 1	ATGCAG	1	1	61	0.875	0.875
Heptamer recombination signal	CACAGTG		-	24		-
Murine MHC recombination hotspot	CAGRCAGR	2	1	16	0.103	0.420
Recombination hotspot	CCNCCNTNNCCNC	1	-	9	0.272	-
DNA polymerase a frameshift hotspot 2	CTGGCG	-	-	5	-	-
Murine parvovirus recombination hotspot	CTWTTY	5	6	294	0.993	0.974
Ig heavy chain class switch repeat 1	GAGCT	4	4	141	0.703	0.703
Translin-binding site 2	GCCCWSSW	2	2	42	0.403	0.403
X-elementæEscherichia coli	GCTGGTGG	-	-	4	-	-
Human minisatellites conserved sequence/X-like element	GCWGGWGG	-	-	15	-	-
Human minisatellites core sequence	GGGCAGGARG	-	-	1	-	-
Ig heavy chain class switch repeat 2	GGGCT	3	5	116	0.754	0.313
Ig heavy chain class switch repeat 3	GGGGT	3	3	94	0.605	0.605
Vertebrate topoisomerase II consensus	RNYNNCNNGYNGKTNYNY	-	-	6	-	-

DNA polymerase a frameshift hotspot 1	TCCCCC	2	3	469	0.2727	0.08109
Ig heavy chain class switch repeat 5	TGAGC	7	4	337	0.2486	0.8172
Ig heavy chain class switch repeat 4	TGGGG	7	6	346	0.2015	0.3726
DNA polymerase a/b frameshift hotspot 2	TGGNGT	4	7	344	0.7847	0.2116
Deletion hotspot consensus	TGRRKM	14	14	84	0.4846	0.4846
Consensus SAR 2	TTWTWTTWTT	0	2	454	1	0.4485
Consensus SAR 4	TWWTDTTWWW	2	5	346	0.9766	0.5804
Consensus SAR 3	WADAWAYAWW	3	4	389	0.7244	0.494
DNA polymerase arrest site	WGGAG	11	12	189	0.4016	0.2211
ARS consensusSchizosaccharomyces pombe	WRTTTATTTAW	-	-	495	-	-
ARS consensusæS. cerevisiae	WTTTATRTTTW	-	-	498	-	-
Vaccinia topoisomerase I consensus	YCCTT	11	9	189	0.4016	0.7739
Ade6-M26	ATGACGT	-	-	498	-	-
Consensus SAR 1	AATAAAYAAA	-	-	491	-	-
D.ætopoisomerase 2 consensus	GTNWAYATTNATNNR	-	-	-	-	-
Human hypervariable minisatellites sequence 1	GGAGGTGGGCAGGARG	-	-	-	-	-
Human hypervariable minisatellites sequence 2	AGAGGTGGGCAGGTGG	-	-	-	-	-
Human replication origin consensus	WAWTTDDWWW DHWGWHMAWTT DHWGWHMAWTT	-	-	-	-	-
LTR-IS motif	TGGAAATCCCC	-	-	-	-	-
Mariner transposon-like element	GAAAATGAAGCTATTTACCCAGGA	-	-	-	-	-
Pur-binding site	GGNNGAGGGAGA	-	-	-	-	-

	RRRR					
Retrotransposon	TCATACACCACGCA GGGGTAGAGGACT	-	-	-	-	-
XY32 homopurine-pyrimidine H-palindrome motif	AAGGGAGAARGG GTATAGGGRAAGA GGGAA	-	-	-	-	-

Supp. Table S5. Description of loci and primers used for microsatellite analysis

Micro - satellite locus	Genomic location	Nature of repeat	Forward primer (5' – 3')	Reverse primer (5' – 3')
Q1	125997870-125997910	CA	TGTAAAACGACGGCCAGTTGTCCTCTGTCTGGAGTCTTTG	TCAAGACCATCCTTGCTAACAC
Q2	126050045-126050082	TC	TGTAAAACGACGGCCAGTCAGCCCCAGATTTGCTTCTAT	TCTGAACAACAGAGCGAGACAT
Q4	126059991-126060030	TG	TGTAAAACGACGGCCAGTGCTGGTGAAGCTGTAGAGGAAT	CACCATGCCCAGCCTATAAT
Q5	126070431-126070475	GAT	TGTAAAACGACGGCCAGTTGAATGAGACTTCCTCCCAAAG	GCAGAGGTTGCATTGATTGAG
Q6	126136644-126136673	TA	TGTAAAACGACGGCCAGTACACAAAAAGGACACCTCTTCC	AACGGAGTGAAACGCTGTCT
Q7	126250460-126250504	AC	TGTAAAACGACGGCCAGTCTAGCAAGCTCTCCCTTCTCTG	GCCGTTAGTGGAGGTCAGTAAG
Q10	126252385-126252425	AC	TGTAAAACGACGGCCAGTGGCCTCCTTCATTTACACTGAG	CTGAGACTGGGTGACTGATGTG
Q8	126267263-126267303	AC	TGTAAAACGACGGCCAGTGGTCCTGACTTCTTGGCTAGTG	AGGCTAGTGATTTCTGGCTTCTAC

Underlined sequences represent the M-13 tag attached to each forward primer.
